# Supplementary material for: Mechanisms governing the pioneering and redistribution capabilities of the non-classical pioneer PU.1
Source: Nat Commun. 2020 Jan 21;11:402. doi: 10.1038/s41467-019-13960-2 (PMC6972792; doi:10.1038/s41467-019-13960-2)
Supplement: Supplementary file 7 — Source data [file 41467_2019_13960_MOESM7_ESM.zip › Source_Data/FigureS2/FigureS2B_MotifScanOutput/KmeansCluster2/homerResults/motif10.similar.html]

motif10

## Information for motif10

A
G
T
C
G
A
C
T
A
G
C
T
A
G
T
C
A
G
T
C
C
G
A
T
A
G
T
C
G
T
A
C
G
A
C
T
A
T
G
C
A
T
G
C
G
C
A
T
A
G
T
C
  
Reverse Opposite:  

T
C
A
G
C
G
T
A
T
A
C
G
T
A
C
G
C
T
G
A
A
C
T
G
T
C
A
G
C
G
T
A
C
T
A
G
C
T
A
G
T
C
G
A
C
T
G
A
T
C
A
G
  

|  |  |
| --- | --- |
| p-value: | 1e-103 |
| log p-value: | -2.385e+02 |
| Information Content per bp: | 1.672 |
| Number of Target Sequences with motif | 685.0 |
| Percentage of Target Sequences with motif | 13.64% |
| Number of Background Sequences with motif | 2021.8 |
| Percentage of Background Sequences with motif | 4.97% |
| Average Position of motif in Targets | 97.8 +/- 50.0bp |
| Average Position of motif in Background | 98.3 +/- 88.4bp |
| Strand Bias (log2 ratio + to - strand density) | -0.1 |
| Multiplicity (# of sites on avg that occur together) | 1.06 |
| Motif File: | file (matrix) reverse opposite |

### Similar de novo motifs found

|  |  |  |  |  |  |  |  |
| --- | --- | --- | --- | --- | --- | --- | --- |
| Rank | Match Score | Redundant Motif | P-value | log P-value | % of Targets | % of Background | Motif file |
| 1 | 0.831 | A G C T A G T C A G T C A G C T A G T C A G T C A G C T A G T C A G T C A G C T | 1e-90 | -209.111883 | 6.71% | 1.49% | motif file (matrix) |
| 2 | 0.755 | G T C A T C G A C A T G A C T G C G T A A C T G A C T G T C G A A C T G A C T G G C T A C A T G A T C G T C G A | 1e-80 | -184.712730 | 5.30% | 1.04% | motif file (matrix) |
| 3 | 0.787 | A G T C A C G T A G T C A G T C A G C T A G T C A G T C A C G T A G T C | 1e-34 | -79.828959 | 5.99% | 2.52% | motif file (matrix) |
